# A Deep Intronic Mutation in the Ankyrin-1 Gene Causes Diminished Protein Expression Resulting in Hemolytic Anemia in Mice

**DOI:** 10.1534/g3.113.007013

**Published:** 2013-10-01

**Authors:** Hua Huang, PengXiang Zhao, Kei Arimatsu, Koichi Tabeta, Kazuhisa Yamazaki, Lara Krieg, Emily Fu, Tian Zhang, Xin Du

**Affiliations:** * Department of Medicine, University of California, San Diego, La Jolla, California 92093; † Division of Periodontology, Department of Oral Bioscience, Niigata University Graduate School of Medical and Dental Sciences, Niigata 951-8514, Japan; ‡ Division of Oral Science for Health Promotion, Laboratory of Periodontology and Immunology, Niigata University Graduate School of Medical and Dental Sciences, Niigata 951-8514, Japan; § Department of Radiotherapy, Beijing ChaoYang Hospital, Capital Medical University, Beijing 100020, China

**Keywords:** ankyrin-1, hemolytic anemia, hereditary spherocytosis, deep intronic mutation, quantitative defect

## Abstract

Linkage between transmembrane proteins and the spectrin-based cytoskeleton is necessary for membrane elasticity of red blood cells. Mutations of the proteins that mediate this linkage result in various types of hemolytic anemia. Here we report a novel *N*-ethyl-*N*-nitrosourea−induced mutation of ankyrin-1, named *hema6*, which causes hereditary spherocytosis in mice through a mild reduction of protein expression. The causal mutation was traced to a single nucleotide transition located deep into intron 13 of gene *Ank1*. *In vitro* minigene splicing assay revealed two abnormally spliced transcripts containing cryptic exons from fragments of *Ank1* intron 13. The inclusion of cryptic exons introduced a premature termination codon, which leads to nonsense-mediated decay of the mutant transcripts *in vivo*. Hence, in homozygous mice, only wild-type ankyrin-1 is expressed, albeit at 70% of the level in wild-type mice. Heterozygotes display a similar hereditary spherocytosis phenotype stemming from intermediate protein expression level, indicating the haploinsufficiency of the mutation. Weakened linkage between integral transmembrane protein, band 3, and underlying cytoskeleton was observed in mutant mice as the result of reduced high-affinity binding sites provided by ankyrin-1. *Hema6* is the only known mouse mutant of *Ank1* allelic series that expresses full-length canonical ankyrin-1 at a reduced level, a fact that makes it particularly useful to study the functional impact of ankyrin-1 quantitative deficiency.

Elasticity is a defining characteristic of the red blood cell (RBC) membrane and depends upon maintenance of a high membrane surface area to volume ratio. Even small reductions in surface area result in sequestration of RBCs from circulation and lysis. The ability of RBCs to maintain their membrane surface area is mediated by the underlying spectrin-based cytoskeleton, which is linked to and supports the lipid bilayer through numerous tethering sites (Bennett 1989; Bennett and Baines 2001). Two macromolecular complexes, the ankyrin complex and the 4.1R complex, provide the “vertical” linkages that tether plasma membrane and cytoskeleton (Mohandas and Gallagher 2008). In the ankyrin-based complex, anchorage protein ankyrin-1 links spectrin tetramers to the cytoplasmic domains of the transmembrane proteins band 3 and RhAG (Bennett 1978; Bennett and Stenbuck 1979a, 1980; Hargreaves *et al.* 1980) and comprises the key determinants of linkage between membrane and cytoskeleton. Deficiencies of the proteins involved in “vertical” interactions, including ankyrin-1, α and β spectrin, band 3, and protein 4.2, lead to unstable lipid bilayers that are prone to release as skeleton-free lipid vesicles, resulting in loss of membrane surface area and spherocytosis (Delaunay 2007; Mohandas and Gallagher 2008; Palek 1993).

Ankyrin-1 mutation is the most common cause of hereditary spherocytosis (HS), accounting for approximately 35–65% cases in Northern European populations (Eber *et al.* 1996; Gallagher 2005; Lanciotti *et al.* 1997). These mutations have been detected across the entire *Ank1* gene. Missense and promoter mutations are common in recessive HS, whereas nonsense, frame shift, and splicing mutations mostly result in dominant HS; *de novo* ankyrin mutations occur with high frequency (Eber *et al.* 1996; Gallagher 2005). The 210-kDa full-length ankyrin-1 protein contains three major functional domains: an N-terminal band 3-binding domain, a central spectrin-binding domain, and a C-terminal regulatory domain containing a death domain motif (Bennett 1992; Peters and Lux 1993; Rubtsov and Lopina 2000). The regulatory domain modulates the affinities of both the spectrin- and band 3-binding domains for target proteins (Hall and Bennett 1987).

Elucidation of the pathogenesis of *Ank1* mutations in HS has benefited from the analysis of ankyrin-1 mutant mice. Four mutant lines have been reported. In the normoblastosis mice (*nb/nb*), spontaneous deletion of a guanosine residue in exon 36 of *Ank1* led to a frameshift and premature stop codon, resulting in production of a truncated hypomorphic protein of 157 kDa (Birkenmeier *et al.* 2003). The truncated protein lacks the C-terminal regulatory domain but maintains band 3- and spectrin-binding domains. In addition, although spectrin levels were reduced to 50% of wild-type levels (Lux *et al.* 1979), expression of other integral membrane proteins was preserved. The *Ank-1^1674^* (RBC2), *Ank1^E924X^*, and *Ank1^MRI23420^* mutations were generated by random germline mutagenesis. *Ank-1^1674^* (RBC2) is a null mutation induced by *N*-ethyl-*N*-nitrosourea (ENU), leading to complete deletion of ankyrin-1 protein in homozygous animals, with a concomitant reduction in spectrin and protein 4.2. As a consequence, RBC2 red blood cells exhibited total disruption of membrane skeleton architecture, and the phenotype was manifested as a recessive trait of severe hemolytic HS in mice (Rank *et al.* 2009). Both *Ank1^E924X^* and *Ank1^MRI23420^* lines are hypomorphic mutations, resulting in truncated ankyrin-1 proteins lacking both spectrin-binding domain and C-terminal regulatory domain, whereas band 3-binding domain remains intact in *Ank1^E924X^* (Hughes *et al.* 2011) or partially affected in *Ank1^MRI23420^* (Greth *et al.* 2012).

Here we report the identification and characterization of a novel ENU-induced mutation in *Ank1* named *hema6*. Both heterozygous and homozygous animals exhibit characteristic features of hereditary spherocytosis, such as increased RBC counts, low RBC mean corpuscular volume (MCV), increased osmotic fragility, and shortened life span of RBCs *in vivo*. The causal mutation resides deep in intron 13 of *Ank1*, causing incomplete alternative splicing of the transcript that preserved the production of wild-type ankyrin-1 protein at a reduced level in homozygous animals. The aberrant splicing isoforms are predicted to encode a truncated protein containing two thirds of the band 3-binding domain. However, the mutant protein could not be detected in the ghost membrane and whole RBC lysate. Thus, *hema6* mutation is distinct from other *Ank1* mutants described previously. The analysis of these mice highlights the importance of optimal ankyrin-1 protein quantity in maintaining erythrocyte membrane stability.

## Materials and Methods

### Mice

Animals were generated on a pure C57BL/6J background and were maintained under standard housing conditions and fed with laboratory rodent diet no. 5001 (LabDiet). Unless otherwise stated, 5- to 12-wk-old female and male mice were used in all experiments. All studies were approved by the Institutional Animal Care and Use Committee at University of California, San Diego.

### Hematology analysis

Whole blood was collected by submandibular bleeding into ethylenediamine tetraacetic acid k3-salt-containing microvette 100 tubes (Sarstedt) and analyzed on Scil abc automatic hematology analyzer. Blood smear was stained with Wright-Giemsa stain. Reticulocytes count was analyzed with Retic-COUNT (Thiazole Orange) Reagent (BD biosciences). Serum erythropoietin was measured using Quantikine Mouse/Rat Erythropoietin ELISA (R&D system, Minneapolis, MN). Serum total bilirubin concentration was measured using Total Bilirubin Reagent (Stanbio Laboratory, San Antonio, TX), and following the manufacturer’s protocol.

### Osmotic fragility test

Blood from 6-wk-old mice was tested within 2 hr after collection. One microliter of whole blood was mixed with 200 µL of NaCl gradients ranging from 0.3% to 0.9%, and then incubated at room temperature for 20 min. The mixture was centrifuged and the supernatant’s absorbance was measured at 540 nm. The hemolysis percent was calculated for each solution and plotted against NaCl concentrations. The degree of lysis in 0.3% NaCl is considered to be 100% and 0% for 0.9% NaCl.

### Red blood cells *in vivo* survival assay

*N*-hydroxysuccinimide-biotin (E-Z link; Thermo Scientific) was suspended in sterile saline at 4 mg/mL and injected intravenously into wild-type and *hema6* mice at a dose of 40 mg/kg body weight. Animals were first bled 36 hr after injection, and then at 2−3 d interval in the first 2 wk, followed by 3- to 4-d intervals in the third and fourth weeks, then once in the fifth week; 5−10 µL of blood was sampled each time. Labeled cells were analyzed by flow cytometry after staining with streptavidin phycoerythrin. The percentage of biotinylated RBCs over time reflects the survival of RBCs from each genotype group.

### Electron microscopy

Blood samples were fixed in 2.5% glutaraldehyde at room temperature, and washed in 0.1 M phosphate with 5% sucrose. Aliquots of the cells were then placed on Thermanox coverslips and incubated at 4° for 1 hr to ensure attachment, followed by fixing in 2% osmium tetroxide. After extensive water wash followed by dehydration in graded ethanol series, the samples were processed through a critical point dryer (Tousimis autosamdri 815) and mounted onto scanning electron microscope (SEM) stubs with carbon tape. The stubs with attached coverslips were sputter coated with Iridium (EMS model 150T S) for subsequent examination and documentation on a Hitachi S-4800 SEM (Hitachi High Technologies America Inc., Pleasanton CA).

### Red blood cell ghost preparation, sodium dodecyl sulfate polyacrylamide gel electrophoresis (SDS-PAGE), and Western blot analysis

Approximately 500 μL of blood was collected by cardiac puncture into ethylenediamine tetraacetic acid k3-salt−containing tubes, and sedimented at 1000*g* in 5 mM NaPi (pH 7.4), 150 mM NaCl solution with 0.75% Dextran T-500 at 4°. Packed RBCs were washed four times in ice-cold, phosphate-buffered saline supplemented with 10 mM glucose and then lysed by ice-cold Mg^2+^ lysis buffer (5 mM NaPi, pH 7.4; 1 mM MgCl_2_, 1 mM dithiothreitol, and protease inhibitor cocktail). Ghosts were pelleted by centrifugation at 39,000g.

RBC ghosts or packed RBCs were resuspended in Laemmli sample buffer and subjected to SDS-PAGE using 4−15% gradient gels. After electrophoresis, proteins were either stained with Coomassie blue R250 or transferred to nitrocellulose membrane for Western blotting. The membranes were incubated with individual primary antibodies as indicated and visualized using SuperSignal West Pico Chemiluminescent reagents (Thermo Scientific). Antibody against full-length ankyrin-1 protein was a gift from Velia Fowler; P89 antibody specifically targeted to the N-terminus of ankyrin-1 was kindly provided by Connie Birkenmeier; band 3, protein 4.2 and β-spectrin antibodies were generously provided by Xiuli An; and actin (1−19) was purchased from Santa Cruz Biotechnology. Film was scanned and bands were quantified with ImageJ.

### Detergent extraction of band 3 from red blood cell ghost

As previously described (Gauthier *et al.* 2011), 50 μL of packed ghosts were mixed with 150 μL of phosphate-buffered saline containing various concentrations of Triton X-100, and incubated at 4° for 30 min. The cell suspension was then centrifuged at 21,000*g* for 20 min at 4°, and the supernatant was subjected to SDS-PAGE followed by Western blot analysis using antibody against band 3.

### Measurement of serum iron levels

Serum was mixed with an acid-precipitating/reducing solution (0.6 M trichloroacetic acid, 0.4 M sodium thioglycollate, 2 M HCl). The samples were then centrifuged, and the supernatant was collected from each sample and added to the chromagen bathophenanthroline (1.5 M sodium acetate, 0.5 mM bathophenanthroline disulfonic salt). The optical density of each sample was measured at 535 nm. Iron standards (Thermo Scientific) ranging from 31.25 to 500 µg/dL were assayed simultaneously.

### Histology and tissue iron staining

Spleen sections from wild-type and *hema6* mice were stained with hematoxylin and eosin. Iron staining was performed with standard Prussian blue staining procedure. Histology images were acquired using Zeiss Axio Observer fluorescent microscope, and Zen pro 2011 image capture system.

### Genetic mapping and whole-genome sequencing

Genetic mapping was accomplished by bulk segregation analysis as previously described (Xia *et al.* 2010), using a panel of 127 single nucleotide polymorphisms across the genome. Once a critical region was established by genetic mapping, candidate genes within the region were sequenced.

Whole-genome sequencing of a homozygous *hema6* mouse was performed on Illumina HiSeq2000 system with Next Generation Sequencing core facility at The Scripps Research Institute.

### *In vitro* pre-mRNA splicing assay

Ankyrin-1 mRNA processing was analyzed using ankyrin minigene assay (Cooper 2005). *Ank1* exons 11−16 were amplified from genomic DNAs of wild-type and *hema6* homozygous mice, respectively, using primers 5′ GTCTGTGGGTGCTTGTTGGTGCT 3′ and 5′ TAGCCAGAAGCAGGTCTGGGAGC 3′. The amplified polymerase chain reaction (PCR) products were cloned into vector pcDNA3.1/V5-His-TOPO (Invitrogen, Carlsbad, CA). HEK293 cells transiently transfected with minigene plasmid were harvested 48 hr posttransfection, total RNA was prepared, and reverse-transcription (RT)-PCR was performed using primers corresponding to T7 promoter/priming site and BHG-Reverse priming site on the vector. Amplification products were subcloned and sequenced.

### Quantitative real-time RT-PCR

Bone marrow total RNA was extracted using TRIzol reagent (Invitrogen) and 1 µg of total RNA was reverse transcribed by RETROscript First Strand Synthesis kit (Applied Biosystems, Carlsbad, CA) using random decamers. Quantitative PCR was performed on a StepOnePlus Real-Time PCR system (Applied Biosystems). The primers corresponding to sequences in exons 25 and 26 of *Ank1* (5′ CCGTTGTGATCCGATCTGAAG 3′; 5′ CACAGGGCTAATGTTGTCTGAG 3′) were used. *Gapdh* (5′ GGTCATCATCTCCGCCCCTTCTGC 3′; 5′ GAGTGGGAGTTGCTGTTGAAGTCG 3′) was used as the invariant control.

### Statistical analysis

Statistical significance was evaluated using the unpaired Student’s *t*-test, and differences with *P* <0.05 were considered statistically significant. GraphPad Prism 5 was used for statistical evaluation.

## Results

### *Hema6*, an ENU-induced mutant phenotype of hereditary spherocytosis with mild hemolysis

In an effort to identify proteins with nonredundant function in erythropoiesis, we established a genetic screen for abnormal red cell production and survival in mutant mice generated by large-scale ENU mutagenesis (Arnold *et al.* 2012; Krieg *et al.* 2011). The genetic screen uses simple hematologic measurements of red cell indices and has identified genetic defects resulting in hemolysis, hemoglobinopathy, and iron-deficiency anemia (Krieg *et al.* 2011, H. Huang, L. Krieg, and X. Du unpublished data). The index *hema6* mouse was detected in the screen by its reduced MCV and mean corpuscular hemoglobin but increased counts of RBCs. Tests for heritability indicated that the *hema6* phenotype is transmitted as a dominant trait (Table 1). Platelet counts and the percentages of different leukocyte populations were comparable between *hema6* and wild-type mice, indicating a restricted defect in the erythroid lineage.

**Table 1:** Red blood cell indices of wild-type, heterozygous, and homozygous *hema6* mice

Strain	RBC, 10^6^/µL	HGB, g/dL	HCT, %	MCV, fl	MCH, pg	MCHC, g/dL	RDW, %
wild-type	9.2 ± 0.3	14.1 ± 0.4	43.2 ± 1.8	47.5 ± 0.5	15.4 ± 0.2	32.6 ± 0.3	16.6 ± 1.0
*hema6*/+	10.3 ± 0.6**	14.6 ± 0.7	43.9 ± 2.6	42.2 ± 0.4**	14.2 ± 0.4**	33.4 ± 0.8*	16.0 ± 0.7
*hema6/hema6*	10.2 ± 0.5**	14.2 ± 0.4	41.5 ± 1.7	40.8 ± 0.8**	13.9 ± 0.4**	34.2 ± 1.1**	16.2 ± 0.6

Automated complete blood counts were obtained for six wild-type mice, five *hema6/*+, and nine *hema6/hema6* mice at 6 wk of age; both male and female mice were used. Results are presented as mean ± SD. RBC, red blood cell; HGB, hemoglobin; HCT, hematocrit; MCV, mean corpuscular volume; MCH, mean corpuscular hemoglobin; MCHC, mean corpuscular hemoglobin concentration; RDW, red blood cell distribution width.

* *P* < 0.05, **P < 0.01, Student’s *t* test. Comparisons of *hema6/*+ and *hema6/hema6* with wild-type mice were made, and the statistical significance was shown in the table.

**Figure 1:**
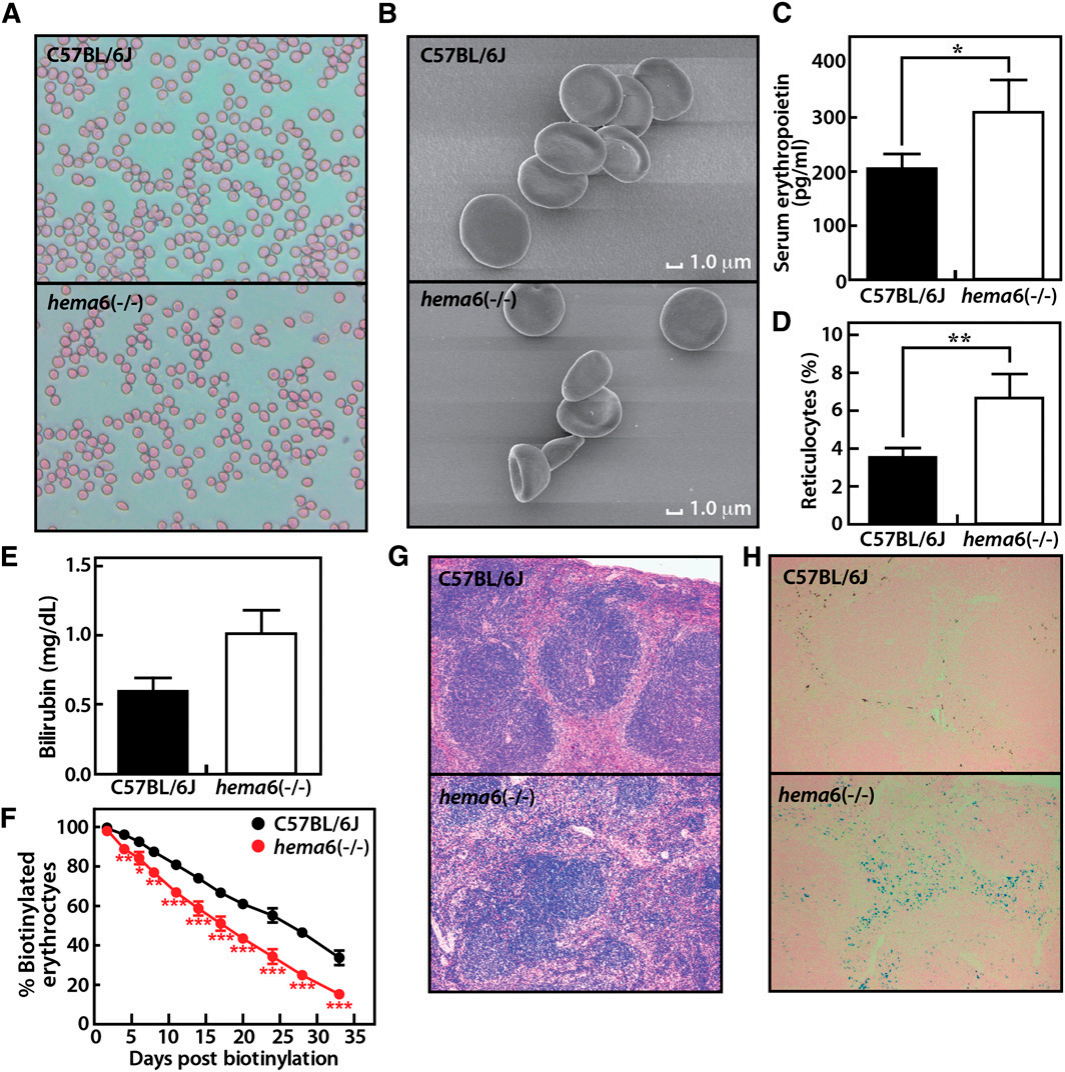
Hematological phenotypes of *hema6* mice. (A) RBCs exhibit spherocytosis when examined by Wright-Giemsa staining of blood smears. Image was taken at 40× magnification. (B) SEM highlighted the morphologic changes in the erythrocytes from *hema*6 mice. (C) Serum erythropoietin level. (D) Reticulocytes count. (E) Serum bilirubin level. (F) Shortened erythrocyte life span in *hema*6 mice. RBCs were labeled with biotin *in vivo* by injecting mice with biotinylation reagent *N*-hydroxysuccinimide-biotin at 6 wk of age. The survival of RBCs was followed by measurement of the percentage of labeled erythrocytes by FACS. In (C−F), measurements were done in mice at 8 wk of age, n = 3 for wild-type C57BL/6J control, n = 4 for *hema*6 homozygotes, data are expressed as mean ± SD. Asterisks denote the level of statistical significance (two-tailed Student’s *t*-test) between *hema6* (−/−) and C57BL/6J mice. *P < 0.05; **P < 0.01; ***P < 0.001. To show the maximum difference between wild-type and *hema6* mice, data on heterozygotes are not shown. (G) Splenic histology from C57BL/6J and *hema6* homozygous mice. Spleens were isolated from wild-type or *hema6* homozygotes and stained for hematoxylin and eosin. (H) High levels of iron deposition in *hema6* (−/−) mice, assessed by Prussian blue staining of spleen sections. *Hema6* mice have much more iron-positive cells (blue straining) relative to C57BL/6J controls. In both G and H, sections are shown at 10× magnification.

Peripheral blood smears showed microcytosis and spherocytosis of *hema6* RBCs (Figure 1A), and SEM highlighted morphologic changes in the erythrocytes from homozygous mice with stomatocytes, spherocytes, and membrane blebbing (Figure 1B). In view of the increased RBC counts, we examined erythrocyte production and destruction in *hema6* mice. Serum erythropoietin level was elevated in *hema6* at 8 wk of age (Figure 1C), indicating an increased erythropoietic activity that possibly accounted for the increased number of mature RBCs; consistently, mild reticulocytosis was observed in *hema6* mice (Figure 1D). Because increased erythropoietic activity may be a compensatory response to premature destruction of RBCs, we evaluated the survival of mature erythrocytes in *hema6* mice. Both homozygous and heterozygous *hema6* mice exhibit elevated total bilirubin concentration in the serum, indicating increased hemolysis (Figure 1E). The survival of erythrocytes *in vivo* was assessed by labeling mature RBCs with biotin and followed the percentile changes of biotinylated RBCs over time. The erythrocyte half-life in *hema6* homozygous mice was approximately 18 d compared with 28 d in the wild-type controls (Figure 1F). The spleens of *hema6* mice were slightly enlarged (97 ± 22 mg in homozygous mutant *vs.* 71 ± 8 mg in wild-type mice), and histology revealed a certain degree of effacement of the normal splenic architecture indicative of extramedullary erythropoiesis (Figure 1G). Iron was excessively deposited in the spleens of *hema6* homozygotes, indicating increased sequestration and destruction of abnormal RBCs (Figure 1H). Liver iron stores was comparable between *hema6* mice and wild-type controls even at 16 wk of age (data not shown).

**Figure 2:**
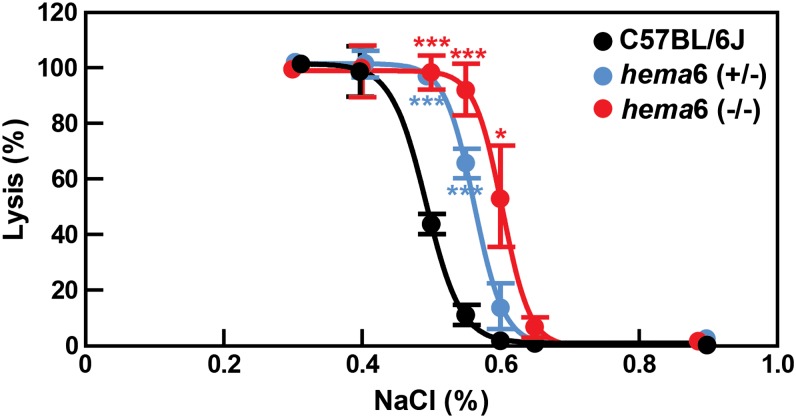
Increased osmotic fragility of *hema*6 RBCs. A total of 1 μL of fresh blood was added to 0.2 mL of a series of hypotonic solutions with NaCl content ranging from 0.3% to 0.9%. The degree of lysis in 0.3% of NaCl is considered to be 100% and 0% for 0.9% of NaCl. N = 3 for both wild-type and *hema*6 heterozygous mice and n = 4 for homozygous *hema*6 mice; data were expressed as mean ± SD. Asterisks denote the level of statistical significance (Student’s *t*-test) between *hema*6 (−/−) and C57BL/6J mice (in red), or *hema*6 (+/−) and C57BL/6J mice (in blue). *P < 0.05; ***P < 0.001.

A characteristic feature of spherocytosis is increased red cell osmotic fragility owing to the decreased surface-to-volume ratio as a result of membrane loss. Consistent with the morphologic changes observed in *hema6* mice, both heterozygous and homozygous *hema6* RBCs exhibited increased osmotic fragility compared to that in wild-type mice (Figure 2).

### Identification of a novel *Ank1* mutation in *hema6* mice

**Figure 3:**
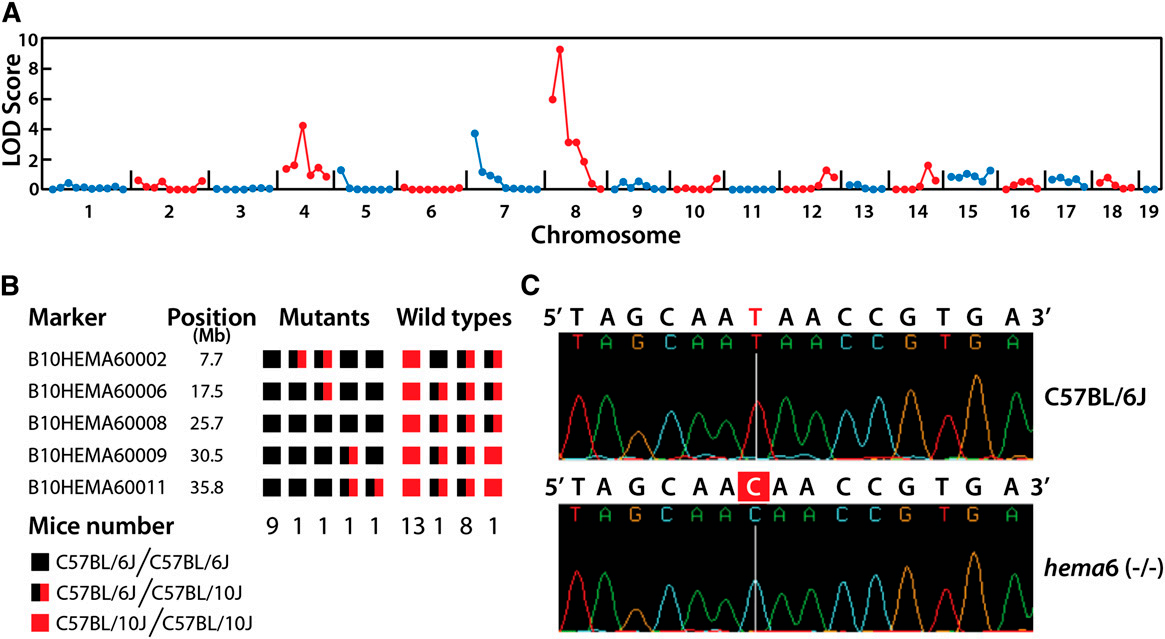
Genetic mapping and positional cloning of phenotypic mutation in *hema6* mice. (A) Genetic linkage on chromosome 8 was established by whole-genome SNPs analysis using 27 mice (13 anemic, 14 nonanemic mice). (B) Results of genotyping individual mice for the C57BL/ 6J and C57BL/10J alleles of the markers on chromosome 8. The number under each column represents the number of F2 mice with the indicated genotypes. The critical region was refined to 13 Mb, between markers B10HEMA60006 and B10HEMA60009. (C) A single nucleotide transition (T>C) was detected in the intron 13 of *Ank1* gene.

To map the *hema6* phenotype, we crossed homozygous mice to wild-type C57BL/10J mice, and the resultant F1 hybrid mice were intercrossed to generate F2 mice for mapping. Thirteen F2 mice displaying anemia (mice with lowest MCV were used) and 14 mice without anemia were used for genetic mapping by bulk segregation analysis, and linkage was observed on chromosome 8 (Figure 3A). Genotyping of individual mice for the C57BL/6J and C57BL/10J alleles of the markers on chromosome 8 confined the critical region containing the *hema6* locus to a 13-Mb interval containing 139 annotated genes (NCBI M37; Figure 3B, Supporting Information, Table S1). Within the critical region, gene *Ank1* encoding erythroid ankyrin-1, whose mutations are the most common cause of typical, autosomal-dominant hereditary spherocytosis (Eber *et al.* 1996; Gallagher 2005; Lux *et al.* 1990), represents a promising candidate. We directly sequenced the genomic locus of *Ank1* in the index *hema6* mouse. Indeed, a homozygous T to C transition was identified in intron13 (Figure 3C), being 791 nucleotides away from exon13 and 209 nucleotides to exon14; the mutation is named *Ank1* IVS13+209T>C, in accordance with the systematic names in nomenclature for mutations in human (Gallagher 2005).

To further confirm IVS13+209T>C is the causative mutation, whole-genome sequencing of a homozygous *hema6* mouse using Illumina HiSeq2000 system was performed. A total of 74% of exonic sequences genome wide was covered at ≥4× coverage. As expected, IVS13+209T>C mutation was detected with 15 high-quality sequencing reads as homozygous variant. Separate genotyping of 60 mice consisting of 22 homozygotes, 31 heterozygotes, and 7 wild-type controls confirmed the concordance between *Ank1^hema6^* genotypes and manifested phenotypes. Therefore, *Ank1* IVS13+209T>C is the causative mutation for the hemolytic hereditary spherocytosis in *hema6* mice.

**Figure 4:**
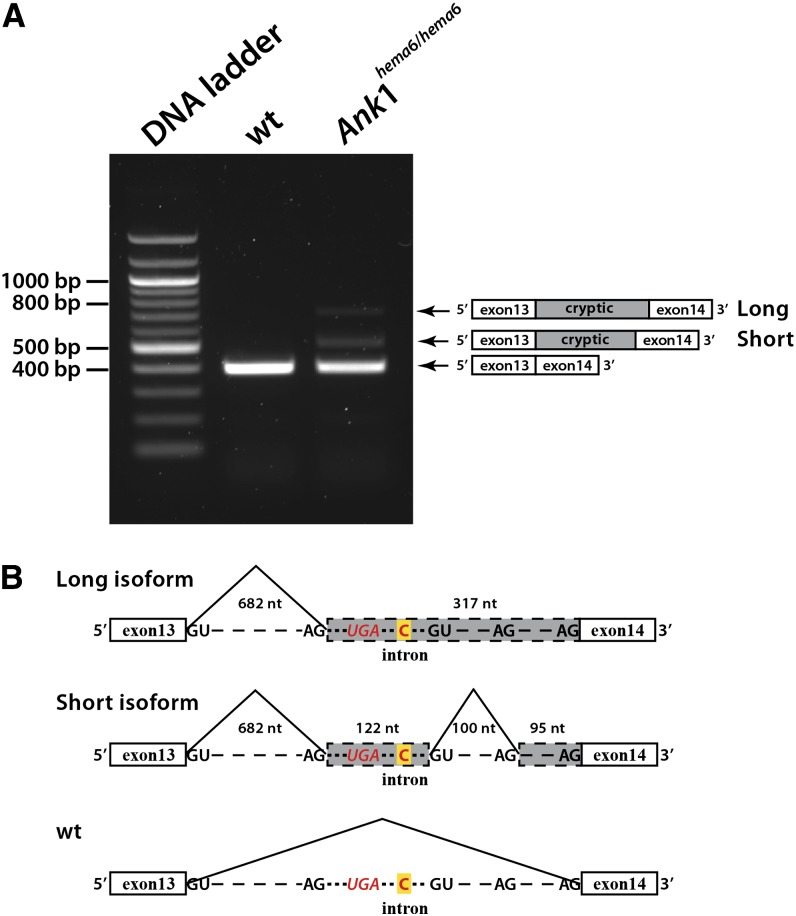
Splicing analyses of *Ank1^hema6^* mRNA transcript. (A) Reverse transcription of *Ank1* mRNA in erythroid progenitors from bone marrow in wild-type and homozygous *hema6* mice. A forward primer located in *Ank1* exon 13 and a reverse primer in exon 14 were used. The transcript structure corresponding to the amplified fragment is shown on the right. (B) Schematic illustration of the splice isoforms resulting from the mutated *Ank1^hema6^* allele. Original exons 13 and 14 are represented by open frame, whereas cryptic exons are shaded in grey (not at scale). The mutated nucleotide residing in the middle of intron 13 is highlight in yellow and shown in bold red font. All 5′ and 3′ splice sites that were activated and used to generate different splicing forms within intron 13 are shown along the line, and the in-frame premature stop codon is indicated in italic red fonts. The sizes of removed introns and cryptic exons in 2 splice isoforms are annotated in the figure.

Intronic mutation can create cryptic splice site that competes with the normal splice sites during RNA processing, resulting in mature mRNA with improperly spliced intron sequences. In order to determine whether *Ank1* IVS13+209T>C is associated with alternative pre-mRNA splicing, we performed RT-PCR with total RNA extracted from bone marrow using primers in exons 13 and 14. As expected, the RT-PCR product of RNA isolated from wild-type C57BL/6J mice yielded an normal cDNA fragment of 400 bp. However, three cDNA fragments were reverse-transcribed from RNA isolated from *hema6* homozygotes: one major segment corresponding to the size of the normal cDNA fragment and two additional fragments of greater molecular weight, but in very low abundance. This finding suggests that they may be aberrant transcripts resulted from alternative splicing (Figure 4A). Therefore, we further analyzed *Ank1* mRNA processing in *hema6* mice by using ankyrin minigene in an *in vitro* functional splicing assay. An *Ank1* minigene corresponding to sequences spanning exons 11−16 was prepared from wild-type and *hema6* homozygous mice and transfected into HEK293 cells, respectively. The pre-mRNA splicing of the minigene was evaluated by analysis of the resulted transcripts. RT-PCR products from HEK293 cells transfected with the wild-type minigene showed one specific band with the expected size, indicating correct mRNA splicing from exons 11 to 16, whereas the *Ank1^hema6^* minigene yielded a correct spliced transcript and the same two splicing isoforms as identified in bone marrow RNA, containing cryptic exons derived from intron 13 sequences (Figure S1). The long splicing isoform contained a cryptic exon of 317nt resulted from the use of a preexisting 3′ splice site that locates 108 nt upstream of *Ank1* IVS13+209T>C mutation; the short splicing isoform incorporated two cryptic exons defined by using aforementioned 3′ splice site and two other preexisting 5′ and 3′ splice sites at downstream of *Ank1* IVS13+209T>C mutation (Figure 4B and Figure S2). Both mutant transcripts would introduce an in-frame stop codon resulting in the premature truncation of protein ankyrin-1, with addition of 8 aberrant amino acids after residue 501. However, we were unable to detect the full-length aberrant mRNA, indicating nonsense-mediated decay of the mutant transcripts.

### *Ank1^hema6^* mutation causes reduction in quantity of ankyrin-1 protein, resulting in weakened interaction between the RBC membrane and cytoskeleton

**Figure 5:**
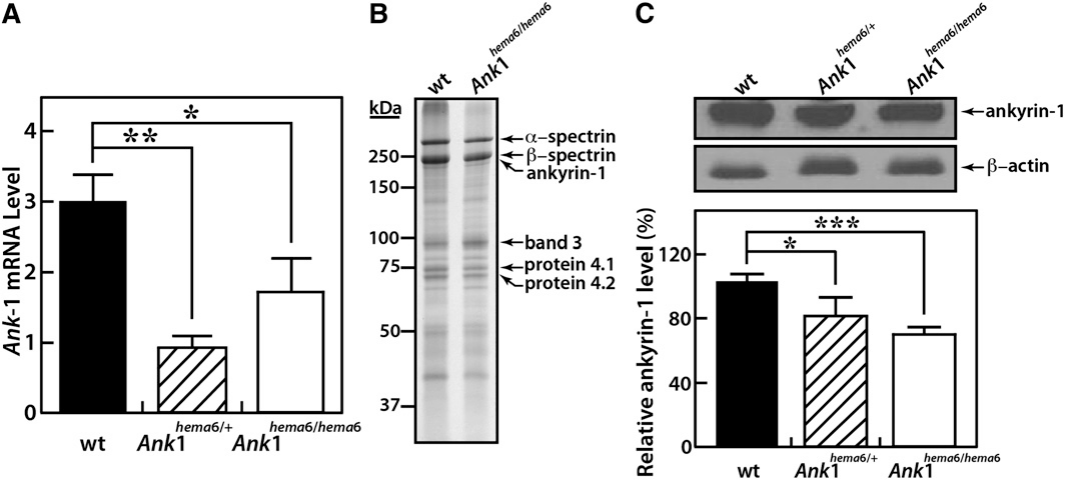
*Ank1^hema6^* mutation causes decreased ankyrin-1 protein expression on the RBC membrane skeleton. (A) Wild-type *Ank1* mRNA level was reduced in *hema6* mice. Total RNA was isolated from bone marrow in both heterozygous and homozygous *hema6* mice, as well as sex- and age-matched wild-type C57BL/6J control mice. *Ank1* expression was assessed by quantitative RT-PCR with GAPDH transcript as endogenous control. N = 4 for all groups of mice. Data are expressed as mean ± SD (B) Coomassie blue-stained SDS-PAGE of ghost membrane proteins. (C) Immunoblot of ghost membrane proteins with antibodies against ankyrin-1 and β-actin, respectively (top). The protein levels of ankyrin-1 were quantitated by densitometry (bottom). Result shown here represents three independent experiments, and data are expressed as mean ± SD, n = 3 for all groups. *P <0.05, **P <0.01, ***P <0.001.

The normally spliced *Ank*1 transcript is dominant in homozygous *hema6* mice, indicating that mRNA processing through the cryptic splice sites activated by *Ank1* IVS13+209T>C mutation was less efficient. However, their competition for the use of splicing machinery may reduce the yield of normal *Ank*1 transcript. We, therefore, further quantified *Ank1* mRNA levels in erythroid progenitors from bone marrow in wild-type and *hema6* mice by quantitative real-time PCR. Overall, *Ank1* transcript was significantly reduced in both heterozygous and homozygous *hema6* mice compared with that in wild-type mice. *Ank1* mRNA level is consistently higher in homozygous *hema6* mice than that in heterozygotes, although their difference is not significant (Figure 5A).

We next examined the consequence of *Ank1* IVS13+209T>C mutation on ankyrin-1 protein expression. RBC ghost membranes were prepared from wild-type C57BL/6J and *hema6* homozygous mice, and the protein content was separated on a 4-15% SDS-PAGE gel and stained with Coomassie blue R250 (Figure 5B). The overall staining pattern of the ghost membrane appeared to be identical between wild-type and *hema6* homozygous mice, so was the relative intensity of bands representing major RBC membrane and skeleton proteins, with slightly “thinner” appearance at ankyrin-1 area in *hema6* homozygotes. We further quantified the levels of ankyrin-1 by western blot analysis using an antibody against the full-length ankyrin protein purified from human erythrocytes. Normal ankyrin-1 protein of 210 kDa was readily detected in both wild-type and *hema6* mice but the protein levels were reduced to approximately 81% and 70% of wild-type level in *Ank1^hema6/+^* and *Ank1^hema6/hema6^* ghosts, respectively, when normalized by the actin level (Figure 5C). The predicted truncated ankyrin-1*^hema6^* is approximately 55 kDa, with complete deletion of β-spectrin binding domain, C-terminal regulatory domain, and one third of membrane-binding domain. Presumably, it should exist as a cytosolic protein.

We conducted immunoblotting on whole blood lysates from wild-type, *Ank1^hema6/+^*, and *Ank1^hema6/hema6^* mice with antibody that we used previously to detect the full-length ankyrin-1 protein. Only a 210-kDa band, corresponding to the full-length protein, could be detected in all samples. We reason that the epitope(s) recognized by the antibody raised against full-length protein may not exist anymore in the truncated protein. We further attempted to detect the truncated protein by P89 antibody, which is specifically targeted the N-terminus (amino acid 183−191) of ankyrin-1 protein. Similarly, other than the 210-kDa band, no band at approximately 55 kDa could be observed in ghost membrane and whole blood lysate samples from *Ank1^hema6/+^* and *Ank1^hema6/hema6^* mice; moreover, the western blot didn’t show any other bands distinct in *hema6* homozygous and heterozygous mice from that in wild-type mice (data not shown). These results are consistent with the prediction that the mutant transcripts are subjected to degradation by nonsense mediated decay pathway, and wild-type ankyrin-1 is the only form of protein expressed in *hema6* RBCs.

Within ankyrin-based multiprotein complex, ankyrin-1 attaches β-spectrin tetramers to the cytoplasmic domain of band 3, thereby provide high-affinity linkage between the plasma membrane and underlying skeletal network. We further evaluated β-spectrin, protein 4.2 and band 3 expression levels in *Ank1^hema6/hema6^* RBCs by western blot analysis and eosin-5′-maleimide staining, respectively. *Hema6* homozygous membranes contained comparable levels of β-spectrin and protein 4.2 relative to C57BL/6J controls (data not shown), but showed 80% of wild-type level in band 3 expression (Figure S3).

**Figure 6:**
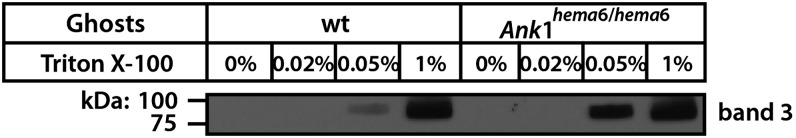
Band 3 extractability from the RBC membrane skeleton in wild-type and *hema6* mice. Ghost membrane proteins were extracted with Triton X-100 at indicated concentration, and proteins released into the supernatant were analyzed by SDS-PAGE and probed with antibody against band 3. Result shown here is representative of three independent experiments.

In normal mouse RBCs, approximately 40% of band 3 molecules are laterally immobilized, and 80% are rotationally immobile or rotate slowly as the result of their association with the cytoskeleton through either high- or low-affinity interactions with ankyrin-1(Rubtsov & Lopina 2000; Yi *et al.* 1997). We hypothesized that reduced quantity of ankyrin-1 would weaken the association of band 3 with cytoskeleton. To evaluate the functional consequence of *Ank1^hema6^* mutation, we performed detergent extraction of band 3 from ghost membrane using Triton X-100. Band 3 was more easily to be extracted from *hema6* ghost membrane than wild-type membrane (Figure 6). Thus, fewer band 3 remained in the membrane of *Ank1^hema6/hema6^* red blood cells than in normal RBCs after detergent extraction, indicating weakened linkage of RBC membrane to the cytoskeleton.

## Discussion

Hereditary spherocytosis is a common hemolytic anemia affecting all ethnic groups; however, its clinical severity varies a great deal, from a mild to severe outcome. Ankyrin mutations are the most common mutations associated with HS and result from qualitative and/or quantitative abnormalities in ankyrin-1 protein. Here, we describe a germline mouse mutant, named *hema6*, that develops mild HS due to a deep intronic mutation in ankyrin-1 encoding gene, *Ank1*, that results in decreased expression of normal ankyrin-1 protein.

Two alternatively spliced transcripts were generated in *hema6* by the use of cryptic splice sites in intron 13 activated by *Ank1* IVS13+209T>C mutation. Pre-mRNA splicing is a highly regulated process that assures the precise excision of introns and ordered array of exons. In addition to the canonical splice-site sequences and branch point, exonic splicing enhancers (ESEs) or silencers (ESSs) also play important roles in the regulation of alternative splicing events, as well as in the definition of constitutive exons (Goren *et al.* 2006; Sironi *et al.* 2004). Subtle changes (*e.g.*, point mutations) in the intronic sequences may enable pseudo exons splicing competent through modulating these elements. Based on ESEfinder and RESCUE_ESE analyses (Cartegni *et al.* 2003; Fairbrother *et al.* 2004), the pathologic substitution of C for T in *Ank1* intron 13 created a novel exonic splicing enhancer motif AA**C**AACCGT, which likely induced the inclusion of cryptic exons encompassing the mutation (Figure S2). Furthermore, the splice-site strength of the cryptic 5′ splice site located 13 nt downstream of *Ank1* IVS13+209T>C was enhanced in *Ank1^hema6/hema6^* compared with that in wild-type *Ank*1 (donor strength score 0.38 in wild-type *vs.* 0.68 in *hema6*), according to the splice-site prediction algorithm SplicePort (Dogan *et al.* 2007).

It is worth mentioning that an exonic splicing silencer motif TTTGGGCA located 12 nt upstream of *Ank1* IVS13+209T>C mutation was identified by both Sironi *et al.* (2004) and Fas_ESS algorithms (Sironi *et al.* 2004; Wang *et al.* 2004). Although the actual impact of the mutation on this ESS element remains to be determined, it is tantalizing to speculate that binding of SR proteins to the novel ESE motif created by the nucleotide transition may pose potential steric hindrance to the binding of hnRNP proteins to ESS, thereby compromising its repression on exonic splicing. Emerging evidences have shown that deep intronic mutations causing subtle changes in pseudo exon sequences can activate their inclusion in mature transcripts and cause genetic diseases, *e.g.*, malignancies and cancers in humans. This highlights the importance of screening for deep intronic mutations in cancer patients, and opens the possibility of antisense gene therapy for the treatment of cancer and other diseases caused by this class of mutation.

*Hema6* erythrocytes underwent premature hemolysis in the spleen but iron stores in the liver was essentially not changed compared with age-matched, wild-type controls. A modest increase of erythropoietin level, reticulocytosis, and extramedullary erythropoiesis in the spleen was observed in *hema6* mice at 8 wk of age, indicating a compensatory erythropoietic response to hemolysis. Excessive iron released by premature destruction of RBCs could be all used to meet the demand of increased erythropoiesis and therefore doesn’t cause apparent iron loading as seen in severe case of hemolytic anemia. Consistently, hematocrit and hemoglobin levels were well maintained in *hema6* mice during long period of time (>6 months). Interestingly, we observed hemolytic anemia worsened in some *hema6* homozygous mice at older age, where a reduction in RBCs number and hemoglobin level became apparent at 7 months of age (Figure S4). Whether the increased hemolysis is due to slow-down of marrow response or further decreased synthesis of ankyrin-1 needs further investigation.

Each erythrocyte contains approximately 10^6^ band 3 molecules, assembled into 4 × 10^5^ intramembrane particles comprising primarily dimers, whereas ankyrin is present in roughly 10^5^ copies per cell, corresponding to a limiting stoichiometry of approximately one ankyrin for four band 3 proteins (Bennett and Stenbuck 1979b; Nigg and Cherry 1980). It is now believed that band 3 tetramers represent the predominant ankyrin binding partners on the red cell membrane (Michaely and Bennett 1995; Thevenin and Low 1990). Accordingly, ~30% reduction in ankyrin-1 in *hema6* homozygous mice would result in ~1.2 × 10^5^ (roughly 12%) band 3 molecules free of high-affinity attachment to the underlying cytoskeleton, and shed off with unstable lipid vesicles. Band 3 expression was consistently preserved at about 80% of normal value in *hema6* homozygotes. The concordance between quantitative reduction of ankyrin-1 protein and phenotypic severity (*i.e.*, reduction in MCV) as witnessed in *Ank1^hema6/+^* and *Ank1^hema6/hema6^* mice further highlighted the importance of adequate ankyrin protein level in stabilizing membrane and maintaining favorable surface area in erythrocytes.

In contrast to existing ankyrin-null or hypomorphic mouse mutants, *hema6* is unique in that only wild-type ankyrin protein was produced in mutant erythrocytes at a modestly reduced level. This finding defect closely parallels some mild human HS cases, such as Ankyrin Napoli and Bugey (del Giudice *et al.* 1996; Morle *et al.* 1997), in which ~24% reduction in overall ankyrin-1 protein level was observed. These patients showed moderate hemolytic anemia at admission and the anemia was compensated during follow-up examination. *Hema6* therefore represents a new model for the trial of preclinical therapeutic interventions for the treatment of HS patients with ankyrin-1 deficiency. Furthermore, it was reported that *nb/nb*, *Ank-1^1674^* and *Ank1^MRI23420^* mice are resistant to malaria infection (Greth *et al.* 2012; Rank *et al.* 2009; Shear *et al.* 1991). However, the contribution of ankyrin-1 to the resistance remains controversial. In *nb/nb* and *Ank1^MRI23420^* mice, it is difficult to evaluate the involvement of wild-type ankyrin-1 in the parasite infection because of the existence of truncated ankyrin proteins. In *Ank-1^1674^*-null mice, severe anemia in the homozygous animals precludes them from the test. In this regard, *hema6* provides a valuable model to evaluate the role of ankyrin-1 in malaria parasites entry and maturation inside the red blood cells.

## Supplementary Material

Supporting Information
