# Supplementary material for: A Deep Intronic Mutation in the Ankyrin-1 Gene Causes Diminished Protein Expression Resulting in Hemolytic Anemia in Mice
Source: G3 (Bethesda). 2013 Oct 1;3(10):1687–95. doi: 10.1534/g3.113.007013 (PMC3789793; doi:10.1534/g3.113.007013)
Supplement: Supporting Information [file supp_3_10_1687__index.html]

A Deep Intronic Mutation in the Ankyrin-1 Gene Causes Diminished Protein Expression Resulting in Hemolytic Anemia in Mice — Supporting Information 

# A Deep Intronic Mutation in the Ankyrin-1 Gene Causes Diminished Protein Expression Resulting in Hemolytic Anemia in Mice

## Supporting Information for Huang *et al.*, 2013

**Files in this Data Supplement:**

- Supporting Information - Figures S1-S4 and Table S1 (PDF, 869 KB)
- Figure S1 - *In vitro* mRNA splicing assay using *Ank1* minigene (PDF, 440 KB)
- Figure S2 - Sequence of exon 13, intron 13 and exon 14 of gene *Ank1* (PDF, 476 KB)
- Figure S3 - Reduced band 3 surface expression in *hema6* mice (PDF, 446 KB)
- Figure S4 - Hemolytic anemia was exacerbated in homozygous *hema6* mice at older age. (PDF, 558 KB)
- Table S1 - SNP markers used to define the critical region on chromosome 8 (PDF, 307 KB)
