# Supplementary material for: A Deep Intronic Mutation in the Ankyrin-1 Gene Causes Diminished Protein Expression Resulting in Hemolytic Anemia in Mice
Source: G3 (Bethesda). 2013 Oct 1;3(10):1687–95. doi: 10.1534/g3.113.007013 (PMC3789793; doi:10.1534/g3.113.007013)
Supplement: Supporting Information [file supp_g3.113.007013_007013SI.pdf]

G3 · Genes | Genomes | Genetics

A Deep Intronic Mutation in the Ankyrin-1 Gene Causes Diminished Protein Expression Resulting in Hemolytic Anemia in Mice

Hua Huang[*], PengXiang Zhao[*], Kei Arimatsu[§,ǂ], Koichi Tabeta[§], Kazuhisa Yamazaki[ǂ], Lara Krieg[*1], Emily Fu[*], Tian Zhang[‡], Xin Du[*]

[*]Department of Medicine, University of California, San Diego, La Jolla, California 92093
[§]Division of Periodontology, Department of Oral Bioscience and [ǂ] Division of Oral Science for Health Promotion, Laboratory of Periodontology and Immunology, Niigata University Graduate School of Medical and Dental Sciences, Niigata 951-8514, Japan
[‡]Department of Radiotherapy, Beijing ChaoYang Hospital, Capital Medical University, Beijing 100020, China

[1] Present address: Department of Pediatrics, Sanford Consortium for Regenerative Medicine, University of California, San Diego, La Jolla, California 92037

DOI: 10.1534/g3.113.007013

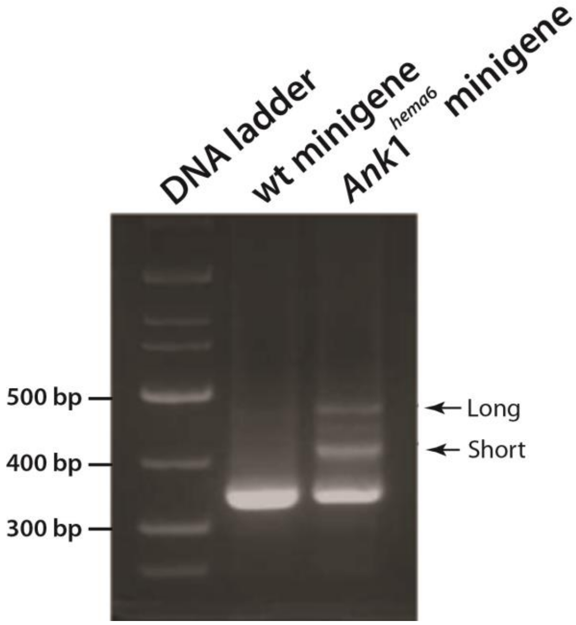

Figure S1 ***In vitro* mRNA splicing assay using *Ank1* minigene.** Wild type and mutant *Ank1*^{hema6} minigenes from exon 11 to exon 16 were prepared and transfected into HEK293 cells. RNA from cells transfected with the wild-type minigene contained the expected normally sized and spliced product, while RT-PCR products derived from RNA of cells transfected with *Ank1*^{hema6} minigene yielded two mutant splice isoforms, in addition to wild type product.

```
Exon 13  AAGCCAACGCCAAGGCCAAG
  1 gtgagctcgg cagacaggga agggagggtc ccaaagagca ggctgcccca  -> authentic splice donor
 51 gcctgatgca agaagccatt ctcatctgca gtctggggct cagagtcatt
101 gatcccccca ttttggtcac actgaaagct caggctctgc tctgttggag
151 ttctgtgagg ggcagcggtc tctttgtatg aagtggtgcc tctatagagt
201 aacagaagcc cagagagcag gctggcccgc agcgtttata gggccagtgg
251 gtagataggg ctgaagggag ggagtccata agatggtacc accagggaaa
301 atcagccagg catcacttga tccagaagag gagcatcctc ttttgtatat
351 tcgatgacaa ataaaccgta agatcagcta ccaagtctgc aagttgacag
401 gctacatggc aaagaccagg tctttgtggg taatggggtc ctttgactcc
451 tctacttgga cccagtaaca gacaccgcat gcgtggagcc cctgtatcgg
501 gagctatagc tggaagttgt agaaaagggc taaatggccg tctctccctt
551 tgggcgattc agagccgcat gactgctgcc acagagcaca aacacaagca
601 taccaggcag aaagagcctg cgagacaggg cttgcttgtc gatagcaaac
651 ggtttgctga gtatcttctc tgaaaccaac agGAACGCCT TGTGTCCATT  -> cryptic acceptor 681
701 AGCTGTTGAT CTGAACCCTA ACCCTATGAA ATAATGTTAC AATTCGCACT
751 TAAACTGAGG AAGCTGAACC TTTGGGCAAT TAAGTAGCAA CAACCGTGAA
801 GCTGGTGAGT TGTAAGGACT GAACTAAGTA GAGCATGTAG TTTCTGTTAA  -> cryptic donor 805
851 GTCAGAGACT GGAAGAAAGT CCAGATCTTT TGATTCATTG TGAGTCACTT
901 GCAGGGTCTG GTCCTCACCT CCTTGCCCTA TGAAATAATA CAGAGTTGGA  -> cryptic acceptor 903
951 CTTGGGAAGG TTAACGTGTC CAGTTTTCTA CTTTCACCCC GTGGCCCAGG
    ATGACCAGACACCGC  Exon 14
```

Figure S2 Sequence of exon 13, intron 13 and exon 14 of gene *Ank1*. Original exon 13 and exon 14 are highlighted in yellow, and the cryptic exons within intron 13 are shown in capital letters. The *hema6* mutation, *Ank1* IVS13+209T>C, is indicated by a bold red font; and donor and acceptor splice sites that were utilized to generate mutant transcripts are indicated by blue and red boxes, respectively. Splice isoform 1 was defined by authentic splice donor and cryptic acceptor 681; splice isoform 2 contained two cryptic exons: one was defined by authentic donor site and cryptic acceptor 681, and the other by cryptic donor 805 and cryptic acceptor 903. The novel exonic splicing enhancer motif is highlighted in green, and the potential exonic splicing silencer element is highlighted in grey.

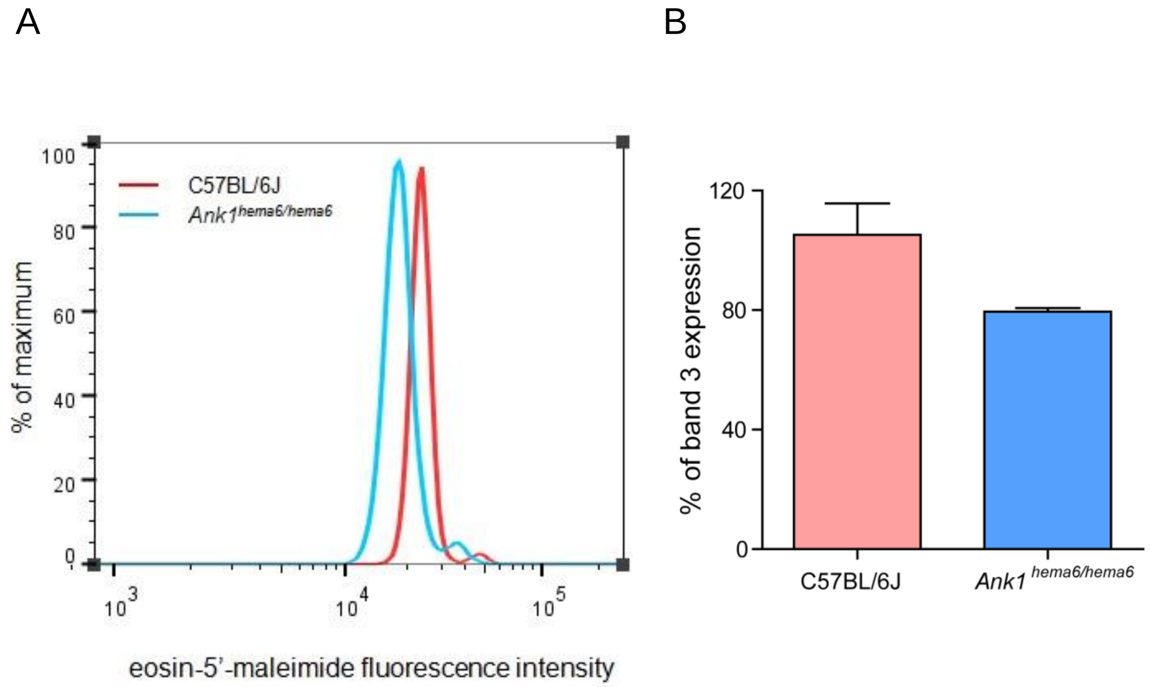

Figure S3 Reduced band 3 surface expression in *hema6* mice. (A) Histogram of eosin-5'-maleimide (EMA) mean fluorescence intensity. Freshly collected blood was washed in PBS and resuspended in EMA solution (0.5mg/ml) and incubated in the dark at room temperature for 1 hour. This suspension was then washed three times with PBS and resuspended in PBS, 0.5% (w/v) BSA, followed by flow cytometry analysis. (B) Quantification of band 3 surface expression based on EMA mean fluorescence intensity. n=4 for both wild type and *hema6* mice, data are expressed as mean ± SD.

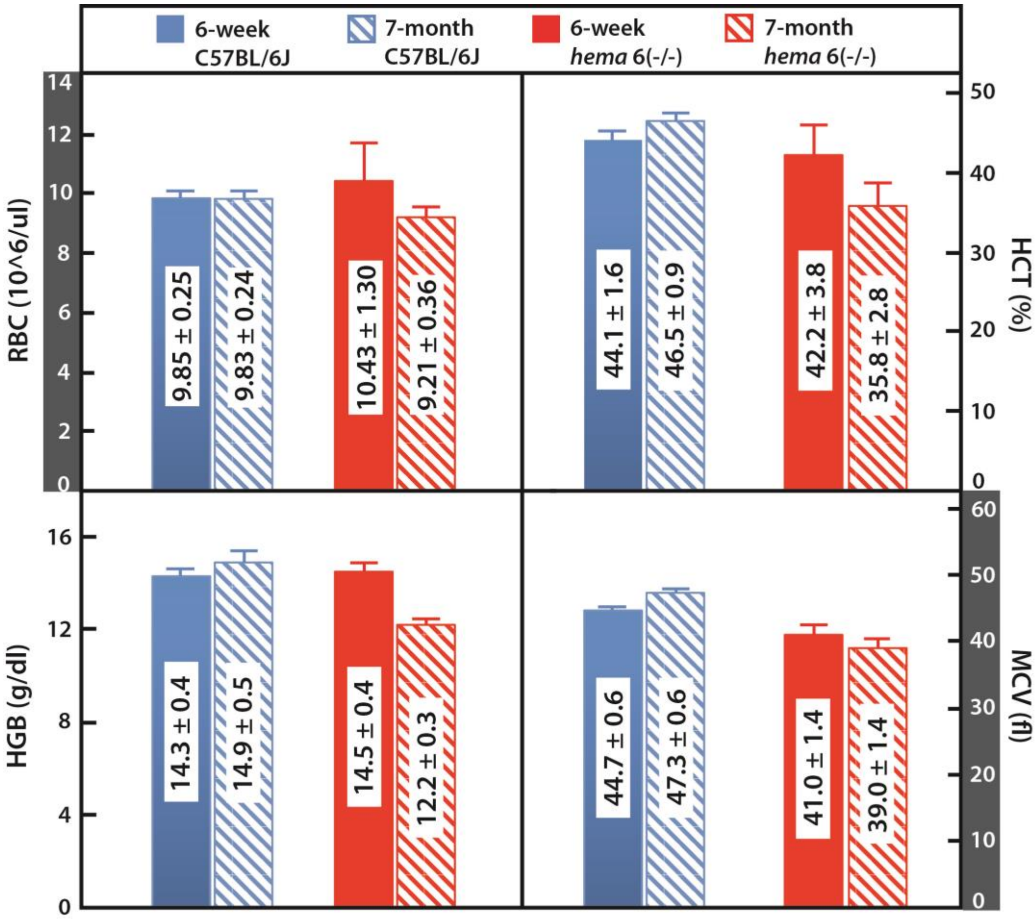

Figure S4 Hemolytic anemia was exacerbated in homozygous *hema6* mice at older age. Red cell indices were analyzed on the same mouse at 6-week and 7-month old of age, respectively for both C57BL/6J and *hema6* homozygotes. n=3 for both groups, and data was expressed as mean ± SD.

Table S1 SNP markers used to define the critical region on chromosome 8

SNP marker	Position (Mb)	B6 allele	B10 allele	PCR ForSeq (5'-3')	PCR RevSeq (5'-3')
B10HEMA60002	7.7	C	G	GTGTAGCAGCTCTGGTTCAGAGATG	GGCCACAGCCAGCATATAGTTTTCC
B10HEMA60006	17.5	G	A	CGTGCCACTAAGACCTCCAGAAATG	TGCTGCTTTAGCAAATAGACCCAGG
B10HEMA60008	25.7	A	G	TCGAAGCTGCCCGTTCTCAATC	ATGGGTCTGAGTTCCTAATTTGCTGAC
B10HEMA60009	30.5	T	C	TCTGCCTCACTGTGAACACAAAGTC	GGCAATCGTCTCTGATGTACTCCAG
B10HEMA600011	35.8	C	T	CAGGTAACTGGAGTTCAAGGTTAGGC	CACTCTTAAAGGTGGGTGAGTCACTG
