# Supplementary material for: A Deep Intronic Mutation in the Ankyrin-1 Gene Causes Diminished Protein Expression Resulting in Hemolytic Anemia in Mice
Source: G3 (Bethesda). 2013 Oct 1;3(10):1687–95. doi: 10.1534/g3.113.007013 (PMC3789793; doi:10.1534/g3.113.007013)
Supplement: Supporting Information [file supp_g3.113.007013_FigureS1.pdf]

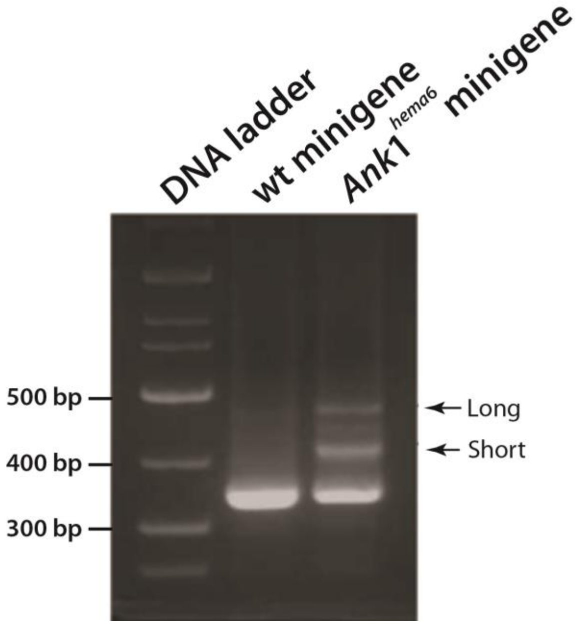

Figure S1 ***In vitro*** **mRNA splicing assay using** ***Ank1*** **minigene.** Wild type and mutant *Ank1*^{hema6} minigenes from exon 11 to exon 16 were prepared and transfected into HEK293 cells. RNA from cells transfected with the wild-type minigene contained the expected normally sized and spliced product, while RT-PCR products derived from RNA of cells transfected with *Ank1*^{hema6} minigene yielded two mutant splice isoforms, in addition to wild type product.
