# Supplementary material for: A Deep Intronic Mutation in the Ankyrin-1 Gene Causes Diminished Protein Expression Resulting in Hemolytic Anemia in Mice
Source: G3 (Bethesda). 2013 Oct 1;3(10):1687–95. doi: 10.1534/g3.113.007013 (PMC3789793; doi:10.1534/g3.113.007013)
Supplement: Supporting Information [file supp_g3.113.007013_FigureS2.pdf]

```
    Exon 13  AAGCCAACGCCAAGGCCAAG
  1 gtgagctcgg cagacaggga agggagggtc ccaaagagca ggctgcccca    authentic splice donor
 51 gcctgatgca agaagccatt ctcatctgca gtctggggct cagagtcatt
101 gatcccccca ttttggtcac actgaaagct caggctctgc tctgttggag
151 ttctgtgagg ggcagcggtc tctttgtatg aagtggtgcc tctatagagt
201 aacagaagcc cagagagcag gctggcccgc agcgtttata gggccagtgg
251 gtagataggg ctgaagggag ggagtccata agatggtacc accagggaaa
301 atcagccagg catcacttga tccagaagag gagcatcctc ttttgtatat
351 tcgatgacaa ataaaccgta agatcagcta ccaagtctgc aagttgacag
401 gctacatggc aaagaccagg tctttgtggg taatggggtc ctttgactcc
451 tctacttgga cccagtaaca gacaccgcat gcgtggagcc cctgtatcgg
501 gagctatagc tggaagttgt agaaaagggc taaatggccg tctctccctt
551 tgggcgattc agagccgcat gactgctgcc acagagcaca aacacaagca
601 taccaggcag aaagagcctg cgagacaggg cttgcttgtc gatagcaaac
651 ggtttgctga gtatcttctc tgaaaccaac agGAACGCCT TGTGTCCATT    cryptic acceptor 681
701 AGCTGTTGAT CTGAACCCTA ACCCTATGAA ATAATGTTAC AATTCGCACT
751 TAAACTGAGG AAGCTGAACC TTTGGGCAAT TAAGTAGCAA CAACCGTGAA
801 GCTGGTGAGT TGTAAGGACT GAACTAAGTA GAGCATGTAG TTTCTGTTAA    cryptic donor 805
851 GTCAGAGACT GGAAGAAAGT CCAGATCTTT TGATTCATTG TGAGTCACTT
901 GCAGGGTCTG GTCCTCACCT CCTTGCCCTA TGAAATAATA CAGAGTTGGA    cryptic acceptor 903
951 CTTGGGAAGG TTAACGTGTC CAGTTTTCTA CTTTCACCCC GTGGCCCAGG
    ATGACCAGACACCGC  Exon 14
```

Figure S2 Sequence of exon 13, intron 13 and exon 14 of gene *Ank1*. Original exon 13 and exon 14 are highlighted in yellow, and the cryptic exons within intron 13 are shown in capital letters. The *hema6* mutation, *Ank1* IVS13+209T>C, is indicated by a bold red font; and donor and acceptor splice sites that were utilized to generate mutant transcripts are indicated by blue and red boxes, respectively. Splice isoform 1 was defined by authentic splice donor and cryptic acceptor 681; splice isoform 2 contained two cryptic exons: one was defined by authentic donor site and cryptic acceptor 681, and the other by cryptic donor 805 and cryptic acceptor 903. The novel exonic splicing enhancer motif is highlighted in green, and the potential exonic splicing silencer element is highlighted in grey.
