# Supplementary material for: A Deep Intronic Mutation in the Ankyrin-1 Gene Causes Diminished Protein Expression Resulting in Hemolytic Anemia in Mice
Source: G3 (Bethesda). 2013 Oct 1;3(10):1687–95. doi: 10.1534/g3.113.007013 (PMC3789793; doi:10.1534/g3.113.007013)
Supplement: Supporting Information [file supp_g3.113.007013_FigureS3.pdf]

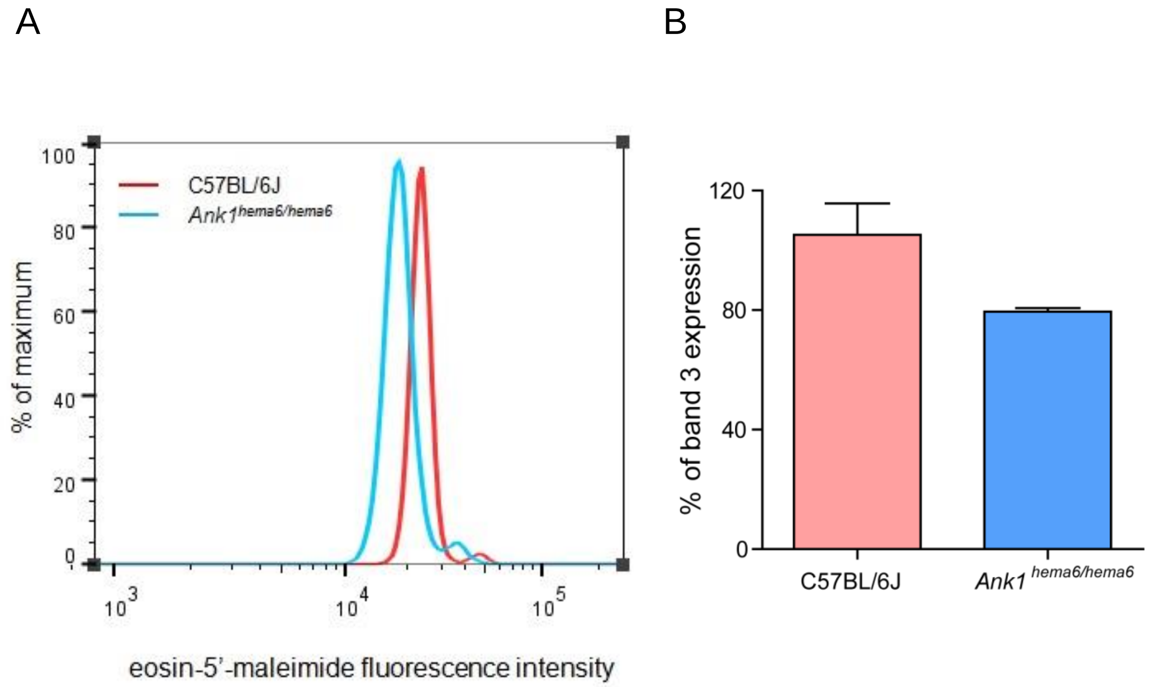

Figure S3 Reduced band 3 surface expression in *hema6* mice. (A) Histogram of eosin-5'-maleimide (EMA) mean fluorescence intensity. Freshly collected blood was washed in PBS and resuspended in EMA solution (0.5mg/ml) and incubated in the dark at room temperature for 1 hour. This suspension was then washed three times with PBS and resuspended in PBS, 0.5% (w/v) BSA, followed by flow cytometry analysis. (B) Quantification of band 3 surface expression based on EMA mean fluorescence intensity. n=4 for both wild type and *hema6* mice, data are expressed as mean ± SD.
