# Supplementary material for: A Deep Intronic Mutation in the Ankyrin-1 Gene Causes Diminished Protein Expression Resulting in Hemolytic Anemia in Mice
Source: G3 (Bethesda). 2013 Oct 1;3(10):1687–95. doi: 10.1534/g3.113.007013 (PMC3789793; doi:10.1534/g3.113.007013)
Supplement: Supporting Information [file supp_g3.113.007013_FigureS4.pdf]

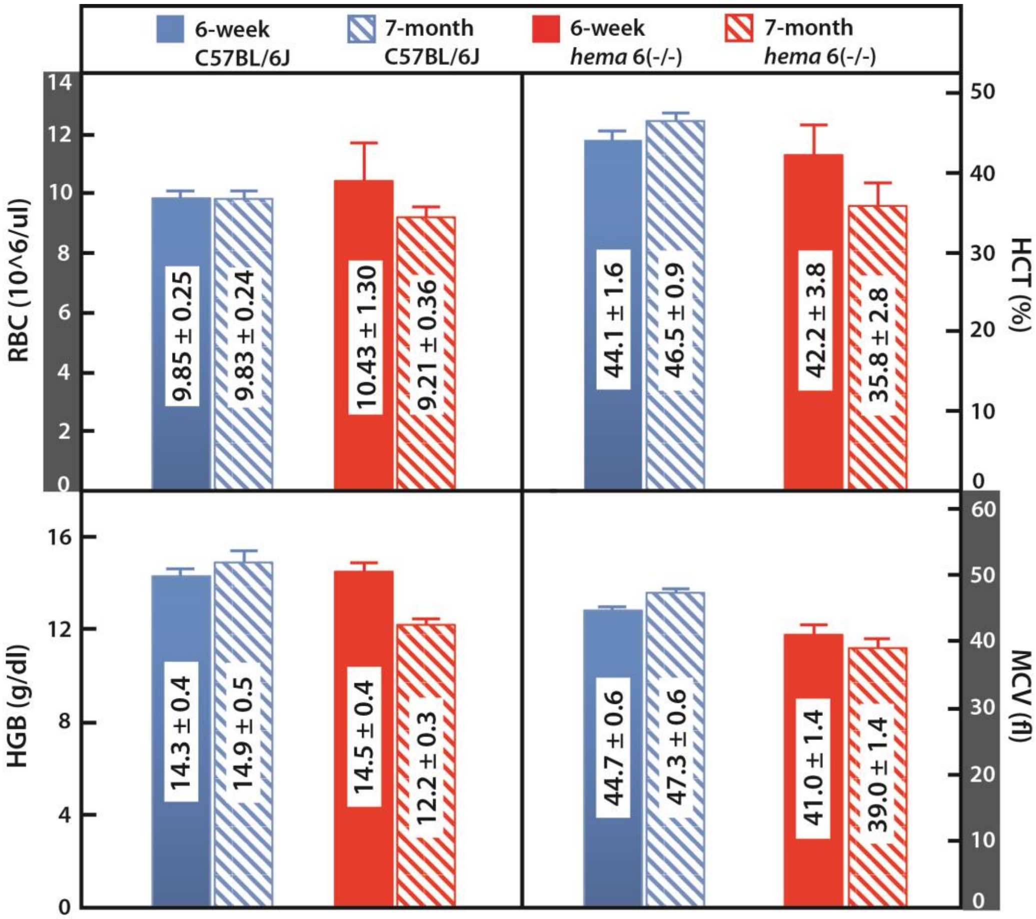

Figure S4 Hemolytic anemia was exacerbated in homozygous *hema6* mice at older age. Red cell indices were analyzed on the same mouse at 6-week and 7-month old of age, respectively for both C57BL/6J and *hema6* homozygotes. n=3 for both groups, and data was expressed as mean ± SD.
