# Supplementary material for: A Deep Intronic Mutation in the Ankyrin-1 Gene Causes Diminished Protein Expression Resulting in Hemolytic Anemia in Mice
Source: G3 (Bethesda). 2013 Oct 1;3(10):1687–95. doi: 10.1534/g3.113.007013 (PMC3789793; doi:10.1534/g3.113.007013)
Supplement: Supporting Information [file supp_g3.113.007013_TableS1.pdf]

Table S1   SNP markers used to define the critical region on chromosome 8

SNP marker	Position (Mb)	B6 allele	B10 allele	PCR ForSeq (5'-3')	PCR RevSeq (5'-3')
B10HEMA60002	7.7	C	G	GTGTAGCAGCTCTGGTTCAGAGATG	GGCCACAGCCAGCATATAGTTTTCC
B10HEMA60006	17.5	G	A	CGTGCCACTAAGACCTCCAGAAATG	TGCTGCTTTAGCAAATAGACCCAGG
B10HEMA60008	25.7	A	G	TCGAAGCTGCCCGTTCTCAATC	ATGGGTCTGAGTTCCTAATTTGCTGAC
B10HEMA60009	30.5	T	C	TCTGCCTCACTGTGAACACAAAGTC	GGCAATCGTCTCTGATGTACTCCAG
B10HEMA600011	35.8	C	T	CAGGTAACTGGAGTTCAAGGTTAGGC	CACTCTTAAAGGTGGGTGAGTCACTG
